# Perceived facilitators and barriers against human papillomavirus vaccine among rural parents with eligible daughters in the Alle district, Southern Ethiopia

**DOI:** 10.1371/journal.pone.0354372

**Published:** 2026-07-22

**Authors:** Selemaye Zenebe, Tomas Yeheyis, Fikru Tadesse, Bedilu Deribe, Gezhagn Geresu, Tsehaynew Kasse, Susanne Unverzagt, Eric Sven Kroeber, Eva Johanna Kantelhardt, Lesley Taylor, Betty Ferrell

**Affiliations:** 1 Department of Midwifery, College of Medicine and Health Sciences, Arba Minch University, Arba Minch, Ethiopia; 2 School of Nursing, College of Medicine and Health Sciences, Hawassa University, Hawassa, Ethiopia; 3 Department of Nursing, College of Medicine and Health Sciences, Arba Minch University, Arba Minch, Ethiopia; 4 Institute of General Practice and Family Medicine, Center of Health Sciences, Martin Luther University Halle-Wittenberg, Halle (Saale), Saxony-Anhalt, Germany; 5 Global and Planetary Health Working Group, Martin Luther University Halle-Wittenberg, Halle (Saale), Saxony-Anhalt, Germany; 6 Institute of Medical Epidemiology, Biostatistics, and Informatics, Center of Health Sciences, Martin-Luther-University Halle-Wittenberg, Halle (Saale), Saxony-Anhalt, Germany; 7 Department of Surgery, Division of Breast Surgery, City of Hope National Medical Center, Comprehensive Cancer Center, Duarte, California, United States of America; 8 Department of Nursing Education and Research, City of Hope National Medical Center, Comprehensive Cancer Center, Duarte, California, United States of America; Federal University of Sergipe, BRAZIL

## Abstract

**Introduction:**

Human papillomavirus vaccine acceptance is influenced by the parental perceptions of the vaccine, but there are few related studies in Ethiopia, and most have focused on quantitative approaches. Therefore, this qualitative study intended to explore parental perceptions about the human papillomavirus vaccine acceptance.

**Objective:**

To explore the perceived facilitators and barriers against human papillomavirus among rural parents with eligible daughters in the Alle district, southern Ethiopia.

**Methods:**

A qualitative study using focus group discussion and in-depth interviews was conducted from April 25, 2023, to May 25, 2023, among eligible parents of daughters in the Alle district, southern Ethiopia. A convenience sample of 53 parents was recruited for focus group discussions, and 15 parents were purposely sampled for in-depth interviews. The data were collected by a semi-structured focus group discussion guide and in-depth interview questionnaires. The interview data were translated into English after transcription, and thematic analysis was done by Atlas software version 7.1.16.

**Results:**

Among the total participants, 66.2% were female parents and 60.2% were protestant religious followers. Findings were split between two major themes: perceived facilitators and barriers to human papillomavirus vaccine acceptance. The perceived facilitators identified were preventing cervical cancer, seeing it as an expression of parental role and responsibility, and believing in the recommendation of health professionals as facilitators of human papillomavirus vaccination. The identified perceived barriers to human papillomavirus vaccination included: a lack of awareness, a lack of reliable information sources, a lack of trust in the vaccine, misconceptions, fear of side effects, and cultural and religious factors affecting the acceptance of human papillomavirus vaccination.

**Conclusion:**

Parents identified various facilitators and barriers to accepting the human papillomavirus vaccine. Therefore, we recommend that the government and concerned institutions work in collaboration to address the identified challenges to improve the acceptance of human papillomavirus vaccination.

## Introduction

Cervical cancer (CC) is the fourth most frequently diagnosed and fourth most common cause of cancer-related mortality among women, accounting for 604,127 new cases and 341,831 deaths globally in 2020. [1] Approximately 90% of cases were in low and middle-income countries (LMICs), with the majority of these nations located in sub-Saharan Africa (SSA), with a higher elevation rate in Eastern Africa. [1,2]

In Ethiopia, one of the East African countries located in SSA, the cervical cancer burden is high and increasing over time. [3] In 2020, the estimated incidence and mortality in the country were 7,500 and 5,340, respectively, and these rates are expected to intensify and double by 2040 due to population growth, aging, and an increase in the prevalence of well-known risk factors. [3,4] These risk factors include sexual activity at a young age, several partners, over two years of oral contraceptive use, smoking, high parity, HIV co-infection, weakened immune system, and sexually transmitted diseases (STDs). [3,5,6] Despite the high burden, the country still has no well-established prevention mechanisms due to the low socioeconomic status of the general population and low health service utilization. [3,7]

Human papillomavirus (HPV) is found in more than 99% of cervical cancer cases. [8] Based on their correlation with cervical cancer and its precursor lesions, the fifteen different types of HPV that have been associated with cervical cancer are categorized as high-risk (oncogenic) or low-risk (non-oncogenic). [9] While types 16, 18, 31, 33, 35, 39, 45, 51, 52, 56, 58, 59, 68, 73, and 82 are high-risk or oncogenic HPVs, types 6, 11, 42, 43, and 44 are low-risk or non-oncogenic HPVs. [8–10]

Prevention is the most long-term, cost-effective strategy for cancer control. [11,12] The development and availability of a vaccine against the human papillomavirus (HPV) have presented an exceptional opportunity for cervical cancer prevention. [13] A key strategy for prevention is HPV vaccination of adolescent girls before sexual contact. [12,14] The main method for lowering the frequency and effects of cervical cancer in low-resource settings, such as Ethiopia, where there is a dearth of screening and diagnostic tools and medical facilities, is primary prevention (vaccination). [15] As a result, Ethiopia adopted the HPV vaccination for teenage girls aged 9–14 in 2018. [16]

Bivalent and quadrivalent vaccines are currently being used in Ethiopia to immunize females against HPV. [17] Girls aged 9–14 years were planned to be vaccinated before the onset of sexual activities. [16] However, due to a global HPV vaccine shortage, the country has been implementing the vaccine in a school-based approach. With the support of the Global Alliance for Vaccines and Immunization (GAVI) since December 2018, a vaccination campaign for a single cohort of 14-year-old girls has been undertaken with the hope of expanding to additional cohorts based on the global availability of the vaccine. [17,18]

Parents’ perceptions and knowledge are the main factors for the acceptance of HPV vaccination. [15] However, lack of knowledge of HPV vaccination and associated misconceptions remain the most challenging factors to the implementation of HPV vaccination in developing nations such as those in SSA. [7] Recent studies show that there are drastic disparities in vaccination rates based on cultural beliefs, even in countries with vaccine education. [11,15,19] Missed vaccination often occurs in the setting of stigmatization of HPV as a sexually transmitted infection. In SSA, lack of awareness, misconception of the HPV vaccine, and fear of pain and adverse events have been reported as the main reasons given by adolescent girls and their parents for not being vaccinated. [7,20]

In Ethiopia, lack of awareness about the HPV, lack of family support for the HPV vaccination, poor perception about the HPV vaccine, fear of side effects of the HPV vaccine, religious considerations, and misunderstanding of the vaccine were among the major factors that influence HPV vaccine uptake among adolescent girls. [21–23] To the best of our knowledge, no qualitative studies have been done exclusively on parents’ perceptions, especially in the rural setting of Ethiopia. Therefore, this study aimed to assess perceived facilitators and barriers against human papillomavirus among rural parents with eligible daughters in the Alle district, Southern Ethiopia.

## Materials and methods

### Study setting and time

This study was conducted in the Alle district, which is one of the administrative units in the Southern Ethiopian Region. It is 650 km from Addis Ababa, the country’s capital, in a primarily rural area. The district is found in the north of the Konso, Southwest of the Gamo, Southwest of the Derashe district, and Northwest of the South Omo zone. It was composed of 17 rural kebeles (the lowest administrative unit). The population of the district is estimated to be 92,298, living in a total of 18880 households to the census conducted by the district Finance and Economic Development in the year 2023.

### Study design

A qualitative study with thematic analysis was used.

### Eligibility criteria

#### Inclusion criteria.

All parents with a daughter aged 9–14 years in the district.

#### Exclusion criteria.

Parents who were critically ill, unwilling to participate, and unable to communicate in interviews, and those who had resided for less than six months in the area were excluded.

### Sample size and sampling procedure

The data were collected from five focus group discussions (FGDs) with 8–12 parents with eligible daughters. In-depth interviews were held with 15 parents. The FGDs were held in five kebeles (one FGD per kebele), and the in-depth interviews (IDIs) included 3 parents per kebele in those five selected kebeles. Purposive sampling was used to recruit eligible parents who could provide adequate information for in-depth interviews with the support of health extension workers (HEW). Convenience sampling was used, primarily relying on the identification of potential subjects by recommendation from HEWs, and assigned to groups that were considered homogeneous in sex and heterogeneous in characteristics and educational level. Two men’s FGDs and three women’s FGDs were held. The sample size was decided based on the data saturation (the point at which participants had no new information or no new data was discovered). [24,25] The average duration was 55 minutes for FGDs and one hour on average for the in-depth interviews. Subjects did not receive any payment for participation.

**Phenomena of interest:** perception of parents of adolescent girls about HPV vaccination.

### Operational definitions

**Parents of eligible daughters**: parents of daughters aged from 9 to 14 years old, which was the acceptable daughter’s age range for HPV vaccination in Ethiopia.

**Perception about the HPV vaccine**: In this study, the perception of the human papillomavirus (HPV) vaccine is defined as the spectrum of beliefs, attitudes, and views that parents have about the vaccine. This covers information concerning the vaccine’s safety and efficacy, as well as thoughts about its necessity.

**Perceived facilitators of HPV vaccine**: The individual’s belief about factors that motivate the acceptance of the HPV vaccine.

**Perceived barriers to the HPV vaccine**: These are the individual’s views about what stands in the way of accepting and using of HPV vaccine.

**Contextual factors affecting the HPV vaccine:** Include the community’s social, cultural, and religious factors affecting beliefs about the HPV vaccine [26].

**Misconceptions regarding HPV vaccine**: Incorrect beliefs, misconceptions, or misunderstandings of the HPV vaccine and its advantages.

### Data Collection procedures and quality assurance

In-depth interviews were conducted in five kebeles (three parents per kebele) among 15 parents. An interview guide was developed based on a review of the existing literature and included considerations specific to the local context. [27,28] Two pilot interviews were conducted, resulting in minor adaptations to the topic guide. The parents were interviewed in private places with female nurses and midwives.

Focus Group Discussions (FGDs) Guide: A semi-structured FGD guide was used to direct the conversation after adaptation from existing literature. [29] The FGDs were conducted by the MSc midwives and assisted by other BSc nurses.

The questionnaires were prepared in English first and translated into Amharic and then back to English to check the consistency of language and thought by language experts. All in-depth interview and FGD information was tape-recorded after consent from each participant.

### Data processing and analysis

All in-depth interviews and FGDs were captured using voice recorders, and each day, field notes were transcribed into the English language by FGD field facilitators and the researchers. The supervisors and principal investigator independently examined the transcripts for accuracy. The data were analyzed through thematic analysis. The study used both inductive and deductive coding to include core components that didn’t fit with the initially prepared framework of the major themes. Major themes were derived based on the objective of the study.

However, subthemes were derived from the text itself through repeated reading. After reading the transcripts, the researchers identified emergent themes and then coded each theme to delineate individual topics identified during the discussions. English-translated transcripts were coded by Atlas software version 7.5.16.

Statements were coded and assigned to the appropriate theme. Once the themes were determined, the transcripts were re-read to ensure that the themes accurately reflected the data. All themes identified were felt to capture the discussions from the in-depth interviews and FGDs. The findings were presented in narratives by thematic areas based on the objective of the study. The quotes included in the results were typical views expressed in each in-depth interview and FGDs to exemplify emergent themes.

### Ethical consideration

Before the study initiation, ethical clearance was obtained from the Institutional Review Board (IRB) of Hawassa University College of Medicine and Health Science, reference number IRB/298/15. Written permission was obtained from the district health offices. Informed written consent from each study participant was obtained after explaining the purpose of the study. Individuals participated voluntarily, and they were free to withdraw from the study at any time. During the interview, privacy and strict confidentiality were maintained. The information collected from this research was kept confidential and stored in files.

## Results

### Sociodemographic characteristics of the respondents

The study included 68 parents. 5 focus group discussions were held with 53 participants, and 15 parents participated in in-depth interviews. The 53 participants for FGDs were selected from representative samples of five kebeles (Gewada, Goroze, Adiss-Oltima, Kolango, and Turuba), which were randomly selected from a total of 17 kebeles. Convenience sampling was used to recruit eligible parents for the discussion, and purposely selected for the in-depth interview based on their data taken from local health extension workers. The criteria for selection were parents who had at least one girl between 9 and 14 years old (eligible for HPV vaccine) and who volunteered to participate in the IDIs, had heard about the HPV vaccine and HPV, and were able to provide adequate information regarding the vaccine and concerns about it in their context. The FGD groups included 2 male parent groups of 19 male parents from Turuba and Adiss Olitima kebele and 3 women groups of 34 female parents from Gewada, Goroze, and Kolango kebeles. The range of participants’ ages was 29–44 years. The majority of the parents, 41 (60.3%), were from Protestant religions, followed by Orthodox Christians. Regarding the level of education, 43 (63.2%) parents had completed the primary level of education (up to grade 8), and 25 (36.8%) parents had a secondary level of education and above (had at least grade 9 certificates up to university degrees).

### Categories of themes

Parents’ interviews were coded into two major themes based on the objectives of the study. These are the facilitators and barriers to accepting the HPV vaccine. For each key theme, subthemes were identified. Facilitators included a desire to prevent cervical cancer and a perception of the parental role and responsibility. Barriers included misconceptions about the HPV vaccine, fear of unknown side effects, lack of trust in the HPV vaccine, lack of reliable information sources, and contextual factors influencing the HPV vaccine were subthemes to barriers of the HPV vaccine.

### Perceived facilitators of the HPV vaccine

#### Benefits of preventing cervical cancer.

In both FGDs and IDIs, the participants expressed that the main facilitator and motivation to accept the HPV vaccine was the understanding that it can prevent cervical cancer. They emphasized that cervical cancer has serious health effects on the lives of women, and they will vaccinate their daughters to minimize the risk of the disease. Conversely, some participants stated the HPV vaccine is not beneficial and that they don’t want to accept it.

*“My daughter will receive the vaccine since it will help to prevent terrible cancer. The government should promote this for better acceptance, as it is useful for daughters.” (F3P9)*

*I am a teacher, and I have a college diploma. I have been advising my daughters in my school to take the vaccine because I know how much women suffer after they are infected with this disease called cervical cancer. (IDI-P12)*

*I heard that the disease is serious and fatal, and I don’t want to see myself and my daughter suffering from such a disease. I want to know more about it to protect my daughter and myself from this disease. (IDI-P2)*

*I don’t believe that the HPV vaccine is effective in preventing cervical cancer. Because the government is sometimes affected by the influences of foreign policies. So it is not such a beneficial vaccine in my view. (F4P8)*

#### Expression of family role and responsibility.

Parents explained that vaccinating their daughters against the HPV vaccine is one of their roles as parents. They believed that as their daughters are younger, the responsibility to protect them from any harm that they may face is theirs, and they will vaccinate accordingly.

A 36-year-old mother from Goroze said*, “I see my daughter’s vaccination as contributing to the health of her future family. So, I will vaccinate her according to the recommended age in the future.” (F1P3)*

*”As a mother, it is my responsibility to vaccinate my daughter. Sometimes she refuses the vaccination that was given in schools due to misinformation from her friends, but as the disease affects her future life, I should have to advise her to get the HPV vaccine.” (F5P7)*

*“I love my daughter, so I should have to do everything she needs for her well-being. She is unable to analyze the information about the HPV vaccine and cervical cancer, I hear from the group here, due to her younger age. So as a father, I convinced her to accept the HPV vaccine as it is available according to the schedule.” (F3P1)*

*“I have two daughters. They should have to get what is important for their health, but I don’t believe that the HPV vaccine is essential for them. It may lead them to early sex. So I should avoid what leads them to something unnecessary as a mother.” (F1P2)*

#### Trust in information and recommendations from health experts.

Most parents in the FGDs and IDIs said that they believe in the information from health experts and recommend that information be provided to the parents. Parents expressed that they believe in the recommendations of the health experts and are open to hearing from them about the HPV vaccines since they have no other trusted information.

*“I trust health experts more than my friends because they are more educated than us about the HPV vaccine. But most of the time, they only give vaccines to our daughters and don’t inform us about the benefits of the vaccines.” (IDI-P4)*

*“Health information? It was from health professionals; we have no access to electricity, television, radio, or other sources as a community. So, where? But our health professionals haven’t provided adequate health information about vaccines.” (F3P2)*

*“In our society, most of us were farmers and had no formal education. So, we have no information about many things, and we only hear from them if we don’t hear from them. So concerned bodies shall work more on it.” (F1P10)*

### Perception of barriers to accepting HPV vaccines

#### Awareness-related barriers.

In FGDs, most participants had no awareness of the HPV vaccine, and some of the participants had information about the vaccine but had no adequate knowledge about the vaccine and its usage. They recommend that the government and concerned bodies should have to work on the awareness creation and health education.

*“Before I said anything, it was the first time I heard what you said about the HPV vaccine; I have information about other vaccines from extension workers. A vaccine is a vaccine, but what is meant by HPV?” (F2P5)*

*“I have information about this vaccine (HPV vaccine) from a friend recently, but I’m not sure what it is or why it’s necessary.”(F5P5)*

*“I don’t know about this vaccine (HPV vaccine). Sometimes I confuse it with HIV, um….is that? I should have to learn more about it.”(F4P2)*

*“I do not know about the HPV vaccine. I hadn’t heard about it before, but now I have heard it, so I will accept it for my daughters, as cervical cancer has serious consequences on women’s health.” (F1P6)*

*“I don’t know the recommended age at which the girls are vaccinated, but I know that cervical cancer can be prevented by the HPV vaccine.”(F5P4)*

Other participants also expressed thoughts about what information they or what they did not understand:

*“It is new for me to hear about the HPV vaccine. So, health experts and concerned government bodies should have to give us with education about this vaccine before misleading information.” (F5P1)*

*“I have information about this vaccine (HPV vaccine) from a friend recently, but I’m not sure what it is or why it’s necessary,” you said. It is beneficial to prevent cervical cancer. So I need to vaccinate my daughters according to their age. (F2P9)*

*“I heard about the vaccine before. I know that the HPV virus is the main cause of cervical cancer.” (I-P7)*

*“HPV infection can occur after multiple sexual intercourses and polygamy. Also, I have information that the HPV vaccine can prevent cervical cancer.” (I-P13)*

*“I don’t know the recommended age of HPV vaccination since I had daughters, but I don’t know when I can vaccinate her.” (F5P3)*

#### Misconceptions about the HPV vaccines.

Some of the parents thought the vaccine was intended to decrease the fertility rate of adolescents in the future, so they felt they could not allow their daughters to accept the vaccine. Other participants believed that the HPV vaccine has disease-causing strains in it, and it was given to cause disease.

*“I don’t believe in HPV vaccines. Because I heard that it may cause infertility in my daughter in the future. Sometimes health professionals do everything requested by the government to stay on their work, so it is better to believe my friend, as they are concerned about me.” (I-P14)*

*“I don’t think this vaccine is safe for my daughter because she doesn’t like any medication. She is also not at risk of this disease, as I know her, so why should she become vaccinated?” (F2P4)*

*“We lived here for a long time, and we never heard the disease you call cancer. So now is this vaccine to cause cancer or to prevent cancer? They always said vaccine… vaccine, and now we are hearing about new disease cases in our society. Therefore, I don’t trust this vaccine (HPV vaccine) and will never allow it for my daughter.” (F3P3)*

*“I hear this vaccine was given in schools currently because my daughters were run out of the class through the window as the nurse and their teacher closed the door to provide them the HPV vaccine. They came home and told me that the vaccine source was the Illuminati (a demonic organization), intended to decrease fertility among young women. So how can I trust and allow them, as I have no adequate knowledge about it?” (F1P9)*

#### Fear of unknown side effects.

The majority of the parents of both the FGDs and IDIs were concerned about the serious side effects of the HPV vaccine. They feared that the HPV vaccine had serious adverse effects rather than its prevention of cervical cancer, and as a result, they didn’t want to accept the HPV vaccine.

*“I think this vaccination may have another intention in that it targets adolescent girls. Maybe to make them less fertile to decrease population growth, as health professionals taught us before, to decrease family size.” (I-P11)*

*“My daughter told me that the vaccine has severe adverse effects. It causes blood disorders and localized persistent pain in adolescent daughters. Therefore, I don’t want to vaccinate my daughters.” (I-P15)*

*“Cervical cancer is a matter of probability in all women. But if the HPV vaccine is once entered into our blood, we cannot remove it from our blood, and I heard rumors that the HPV vaccine can cause infertility. So how can a woman live without bearing a child in her life in our society? Therefore, I cannot vaccinate with the HPV vaccine for my daughter.” (F4P1)*

#### Lack of trust in the HPV vaccine.

Parents expressed that they don’t trust the HPV vaccine and don’t want to accept it.

*“I am not ready for now to say anything about this vaccine. I may think about it in the future, as I don’t feel confident about it.” (F2P8)*

*“Most vaccines are imported from other countries, and how can we believe it? Especially since we are from a rural population and don’t have enough knowledge about many things. So we should have to think twice about it.”(F5P2)*

*“I have two adolescent daughters, but I never want to allow them to take the HPV vaccine as I have no trust in it.” (F1P7)*

#### Lack of reliable information sources.

The parents expressed that they have limited access to information sources. As they are from rural society, the main information source was person-to-person communication. Also, as the area is rural, they have less chance even to meet health experts to check which information is right.

*“I heard the information about vaccines from my friends. There is no other source of information to hear about the HPV vaccine and its health benefits. During immunization, we vaccinate our children without awareness about it.” (F4P6)*

*“I have no television or radio in my house. I get this type of information from my friends, but for now, I haven’t heard about this vaccine (the HPV vaccine).” (F5P8)*

*“We are far from health institutions, and we meet the health professionals only during vaccine campaigns, or we go there to seek help for our family members. So most of the time, we hear from the community that this vaccine is not good. So I don’t want to allow it to my daughter.” (I-P3)*

*“We are exposed to the rumors in society. Rumors always have some truth behind them. So, I should have to consider which information is real.” (I-P5)*

#### Contextual (cultural and religious) factors influencing the HPV vaccine.

In the FGDs and IDIs, parents expressed their views that taking an HPV vaccine was an expression of a lack of faith, according to their religion. So they shall pray rather than take the vaccine. Other participants think that the HPV vaccine is a foreign thing, and it may affect their chance of marriage, as the man cannot choose them due to the rumors about the vaccine in society.

*“There are different rumors about the vaccine, you say. If a daughter takes the vaccine, it may affect her relationship in the future. Because any man wants to have a relationship with a female whose life is free from any rumors in our culture. Some people believe you can become infertile, and others say you will become a member of the Illuminati. Both conditions are unacceptable in our culture.” (I-P6)*

*“I think being ill is a matter of chance, and God will not take or not take the HPV vaccine. Just leave it for me; I don’t want to allow it to my daughter; rather, I pray for her.” (F5P6)*

*“I have information about the cancer of the cervix and the vaccine that prevents it from the health professionals at the last time campaign. Our society thinks that what we know is another thing. But in my opinion, the government has been giving us lots of vaccines that were important for our children before. So, how can it allow unnecessary things for us now? But thoughts about this vaccine in our society made me not fully trust it. So I never allow my daughter to take it, as I am not ready to recommend it to others about things I don’t fully believe in.” (I-P5)*

## Discussion

This qualitative study intended to investigate the perceived facilitators and barriers against human papillomavirus among rural parents with eligible daughters in the Alle district, southern Ethiopia. Facilitators and barriers to HPV vaccination were identified as two major themes from the study. The perceived facilitators of HPV vaccinations included parents who believe that the HPV vaccine prevents cervical cancer, seeing it as an expression of parental role and responsibility, and believing in the recommendation of health professionals. The barriers to HPV vaccination include a lack of awareness, a lack of reliable information sources, a lack of trust in the vaccine, misconceptions, fear of side effects, and cultural and religious factors affecting the HPV vaccination.

Knowing that the HPV vaccine prevents cervical cancer was taken as the principal motivation for parents to accept the HPV vaccination for their daughter. Parents who believe that the HPV vaccine will prevent cervical cancer in their daughters expressed their willingness to vaccinate their daughters. This finding was consistent with findings from different settings. [29–31]

Parents are also motivated by the fact that vaccinating their daughters against HPV infection is their responsibility, and they contribute to their future lives as responsible parents. They also believe that making their daughters get recommended therapies, such as vaccines, is a way of expressing love. This finding was consistent with a study from Singapore in which participants indicated that parental encouragement to receive the HPV vaccine was often framed in the context of safeguarding long-term reproductive health outcomes. [30]

In addition to the above, trust in information and recommendations from health professionals was reported as one of the facilitators of HPV vaccination. This can be taken as a facilitator to the HPV vaccine because they get accurate information about the benefits of the vaccine. Other findings were that HPV information from health professionals is reliable. [32] Parents repeatedly state that they trust the information from health experts because they think they are more knowledgeable about the vaccines and expect recommendations and adequate information from them in the case of new vaccines. This study is consistent with findings from Malawi, where female parents reported a high level of trust in doctors’ and hospital administrators’ recommendations, stating that they would accept the vaccine for their daughters even if it were newly introduced. [33]

Regarding the barriers to HPV vaccine acceptance, parents reported that a lack of adequate knowledge is the main barrier. Parents thought they should be aware of the vaccine before allowing it for their daughters. This finding is consistent with a study conducted in Wolaita and Addis Ababa, Ethiopia, in which participants reported a substantial awareness gap that needed to be addressed to improve vaccine acceptance. [23,34] However, a study in Nigeria reported that the unavailability of vaccines is the main barrier to the HPV vaccine. [35]

In this study, misconceptions, lack of trust in the vaccine, and fear of side effects were also reported as barriers to the HPV vaccine. Parents mentioned that the HPV vaccine can cause infertility or reduce the ability to become fertile. Others also thought that it may cause blood disorders; some reported that the government was influenced by foreign policies to vaccinate their daughters. This finding is consistent with previous findings from different parts of the world. [23,29,30,34]

This study was conducted among a rural population in Ethiopia, where health literacy was expected to be low. Participants in focus discussions reported that they had no access to mass media due to their residence, and their only trusted source of information was health professionals. However, they also stated that they are exposed to rumors before receiving reliable information, which hinders their acceptance of the vaccine. Participants recommended that the concerned bodies should collaborate to remove this barrier since it affects the implementation of the HPV vaccine. This finding is supported by a previous study in Washington, USA, among immigrant mothers from East African countries. [29]

Cultural and religious factors in this study were reported as barriers among the parents to accepting the HPV vaccine, consistent with the findings from Washington, USA. [29] This study found that accepting HPV vaccination can be interpreted as a lack of faith and should not be recommended. Some participants stated they prefer praying to their creator rather than placing their future in the hands of vaccines. Others reported that vaccinated daughters can be mistreated in their relationships due to rumors arising in society and stigmas associated with the vaccine. This finding was reported by a previous study in Hong Kong. [36] Participants recommended that health professionals should give adequate education to the community to address stigmas about the vaccine and prevent missed treatment against HPV-vaccinated daughters.

### Conclusion and recommendations

Misconceptions, lack of trust, low access to accurate information, and other cultural barriers resulted in low parental understanding and acceptance of the HPV vaccine. As a result, health organizations, concerned bodies, and other local stakeholders must conduct community-based health education and collaborate with local media groups to raise awareness. Other non-governmental organizations, global partners, and stakeholders should suggest and support efforts aimed at improving parents’ awareness and addressing misconceptions. Importantly, public health strategies should also leverage parents’ positive perceptions of the benefits of the HPV vaccine, as these were shown to be directly associated with increased acceptance. Equipping health professionals with the most recent information can further empower them to effectively engage communities and address barriers to vaccine uptake.

## Abbreviations

CC: cervical cancer
FGDs: focus group discussions
GAVI: Global Alliance for Vaccines and Immunization
HPV: Human Papillomavirus
IDIs: In-depth Interviews
IRB: Institutional Review Board
LMICs: low-income and middle-income countries
SSA: sub-Saharan Africa.
