# Supplementary material for: Perceived facilitators and barriers against human papillomavirus vaccine among rural parents with eligible daughters in the Alle district, Southern Ethiopia
Source: PLoS One. 2026 Jul 22;21(7):e0354372. doi: 10.1371/journal.pone.0354372 (PMC13390824; doi:10.1371/journal.pone.0354372)
Supplement: Attachment — Submitted filename: Response to Reviewers.docx [file pone.0354372.s002.docx]

Dear Academic Editor,

We are deeply grateful for the invaluable comments and recommendations provided by you and the reviewers. Your insights have significantly improved the quality and relevance of our manuscript, and we have carefully addressed each point raised. We have highlighted the changes made in the revised manuscript and attached a point-by-point response to the reviewers' comments as requested. Your expert guidance and dedication throughout the review process have been instrumental in enhancing the overall rigor of our work.

Thank you for your thoughtful review and support.

Warm regards,

Selemaye Zenebe, corresponding author

Arba Minch University, selemayez@gmail.com

**Response to reviewer #1 comments**

**Comment 1:** Overall, I think the research was well executed and is about an extremely important topic. It’s very exciting to read about research from Southern Ethiopia and the manuscript will be of interest to an international audience. I have only minor comments.

**Response 1:** Thank you for your insightful feedback. We appreciate your acknowledgment of the relevance of our work.

**Comment 2:** Data availability statement: I think more clarity is needed here. You state “Yes - all data are fully available without restriction” – would this be anonymized or de-identified data? Are all data underlying the findings in their manuscript already available in the manuscript?

**Response 2:** Thank you for your feedback. Deidentified data were used. All the underlying data are available in the manuscript.

**Comment 3:** Abstract: the gender ratio of the participants and the religions of the participants would be of interest to the reader here.

**Response 3:** Thank you for your valuable suggestion. We have added the gender ratio and religion of the participants in the abstract on page 2, line 48.

**Comment 4:** Page 3, line 52: “Cervical cancer (CC) is the ninth most common and also the ninth most frequent cause of new deaths among overall cancer cases. (1)” Is it the ninth most common incident type of cancer and the ninth most common cause of death by cancer? The way this is written, it could be the ninth most common cause of death by cancer and also the ninth most frequent cause of death by cancer (and these are the same). Also, as cervical cancer doesn’t affect men, is it better to delete this sentence and open with the second sentence (which is about a female-only sample)? The rest of this paragraph contains really important information**.**

**Response 4:** Thank you for your feedback. The first sentence was deleted and the paragraph opened with the second sentence in On Page 3, line 63.

**Comment 5:** Page 3, line 71 “These rates are expected to intensify and double by 2040.” – can you let the reader know why?

**Response 5:** Thank you for your feedback. The reasons why the rate will intensify and double by 2040 are updated in the revised manuscript on Page 3, line 70.

**Comment 6:** Page 5, line 120: “A qualitative study was used.” Can you provide more information – e.g. thematic analysis?

**Response 6:** Thank you for your feedback. We added information about the analytical method on Page 5, line 120.

**Comment 7:** Throughout the manuscript, you refer to “conveniently sampled” – this is called ‘convenience sampling’ – e.g. convenience sampling was used. Can you let the reader know how this worked? E.g. flyers, leaflets, the suggestions from health extension workers? Were participants offered a reward for taking part in the study?

**Response 7:** Thank you for your feedback. The researchers used convenience sampling with subjects referred by health extension workers and the participants were not paid to participate explained on Page 5, line 133, and page 5, line 139.

**Comment 8:** Page 5, line 134: “groups that considered homogeneity in characteristics and educational level” – so did you try to get all the groups to be as similar as possible to one another? Why? Often in qualitative research, we aim for heterogeneity (to have diversity of opinion).

**Response 8:** Thank you for your feedback. Page 5, line 134: For FGD the participants were assigned to groups that were considered homogeneous in sex and heterogeneous in characteristics and educational level. The groups were organized into two male groups and three female groups (expressed on Page 5, lines 134&135) with heterogeneity in other socio-demographic aspects. This was because of the cultural views of the society and the women could not freely express their ideas in front of the male parents.

**Comment 9:** Page 6, line 160: “A semi-structured FGD guide was used as a guide during moderation”: I’m not sure what this means – ‘a guide was used as a guide’ – maybe this text can be simplified. What is moderation in this context?

**Response 9:** Thank you for the feedback. The sentence was simplified to: Focus Group Discussions (FGDs) Guide: A semi-structured FGD guide was used to direct the conversation after adaptation from existing literature on page 6, line 162 in the revised manuscript.

**Comment 10:** Page 6, line 170: “The data was analyzed” – the data were analyzed**.**

**Response 10:** Thank you for the feedback**.** Page 6, line 171 on the revised manuscript: “Was” was replaced by “were”.

**Comment 11:** Page 6, line 170: “Major themes were derived based on the objective of the study. However, subthemes were derived from the text itself through repeated reading.” – so it seems that you used a mixture of inductive and deductive coding. If you agree, it would be helpful for readers to know this and you could put it at the start of the study design section, for example.

**Response 11:** Thank you for the feedback. Yes in our study we used both inductive and deductive coding to include core components that don’t fit with the initially prepared framework of the major themes. We added it to the revised manuscript on the method section page 7, line 172.

**Comment 12:** Page 7, line 197: “purposely selected for the in-depth interview based on their data taken from local health extension workers.” Can you tell us about the criteria you used to determine the purposive sampling – e.g. vaccine refusal? Strong beliefs? Verbally skilled? How many criteria were used?

**Response 12:** Thank you for the feedback. Page 7, line 197: “The participants were purposely selected for the in-depth interview based on their data taken from local health extension workers. The criteria for selection were addressed on page 7, lines 200-203 of the revised manuscript.

**Comment 13:** Page 7, line 201: “Regarding the level of education, 43 (63.2%) parents had completed the primary level of education, and 25 parents had a secondary level of education and above” For international readers, it would be useful to have clarity about the age at which these levels of education are typically achieved.

**Response 13:** Thank you for your feedback. We think it is difficult to put a specific age at which the education level was achieved. Since it may be achieved at different ages according to access to education, the time someone started school, school drop rates, and the like. Different studies in our nation reported like ours to specify their education level as primary(grade 1-8), secondary education (grade 9-12), and above means college certificates up to university degrees. We added supportive information on page 8, lines 207 – 209 of the revised manuscript.

**Comment 14**: Page 8, line 204: “Two categories of major themes were identified from the parents' interviews. These are the facilitators and barriers to accepting the HPV vaccine.” This statement contradicts earlier information that told us that the major themes were determined by the objectives.

**Response 14:** Thank you for your feedback. Page 8, line 204: The two major themes were identified based on the objectives but the subthemes for each major theme were identified based on the parent's interviews. These two methods were used to include core components that don’t fit with the initially prepared framework of the major themes from the objective. So we corrected it accordingly and found it on page 8, line 212 of the revised manuscript.

**Comment 15:** Page 15, line 422: “Misconceptions, lack of trust, and different barriers resulted in extremely low parental understanding and acceptance of the HPV vaccine”. This seems to contrast with page 9, line 247: “Most parents in the FGDs and IDIs said that they believe in the information from health experts 248 and recommend that information be provided to the parents. Parents expressed that they believe 249 in the recommendations of the health experts and are open to hearing from them about the HPV 250 vaccines since they have no other trusted information.” How would you characterize extremely low parental acceptance? What were the vaccine acceptance rates? Is that information available?

**Response 15:** Thank you for your feedback. We have updated it and also we have added justification here for the reviewer. Page 15, line 422: it may seem to contrast with page 9, line 247: but the idea from page 9 line 247 refers to the fact that the parents trust the recommendations and information from health experts but they are also exposed to other sources of information including misconceptions, lack of trust in the HPV vaccine and others treatments which resulted in an unwillingness to vaccinate their daughter’s. There was no information available on the actual rate of vaccine acceptance.

**Comment 16:** Page 15, line 424 – do you think addressing misinformation should be a key recommendation from the paper? For example, the concerns about outside interference (the influences of foreign policies), the vaccine is unnecessary, reducing fertility, and being used to control the birth rate, confusion about HPV and HIV, and uncertainty about what cancer is – do these rumors need targeting head on? Are there ways of preparing health workers to have these conversations (in addition to communicating about the benefits)?

**Response 16:** Thank you for your feedback. Page 15, line 424: We agree with your observation that there is a need to dispel these misconceptions. Addressed on page 16, line 430 in the revised manuscript.
