# Supplementary material for: Perceived facilitators and barriers against human papillomavirus vaccine among rural parents with eligible daughters in the Alle district, Southern Ethiopia
Source: PLoS One. 2026 Jul 22;21(7):e0354372. doi: 10.1371/journal.pone.0354372 (PMC13390824; doi:10.1371/journal.pone.0354372)
Supplement: Attachment — Submitted filename: Response_to_Reviewers_auresp_2.docx [file pone.0354372.s003.docx]

Dear Academic Editor,

We are deeply grateful for the invaluable comments and recommendations provided by you and the reviewers once again. Your insights have significantly improved the quality and relevance of our manuscript, and we have carefully addressed each point raised. We have highlighted the changes made in the revised manuscript and attached a point-by-point response to the reviewers' comments as requested. Your expert guidance and dedication throughout the review process have been instrumental in enhancing the overall rigor of our work.

Thank you for your thoughtful review and support.

Warm regards,

Selemaye Zenebe, corresponding author

Arba Minch University, [selemayez@gmail.com](mailto:selemayez@gmail.com)

**Changes were made to the reference list based on the suggestions and comments from the editor and reviewers**

**Retracted references that are removed from the manuscript include:**

1. Cancer WIAfRo. Cancer Tomorrow: estimated number of cervical cancer incidence and mortality in Ethiopia.

2. Organization. WH. Ethiopia launches Human Papillomavirus Vaccine for 14-year-old Girls, 2018 World Health Organization. 2021

**Newly added 8 relevant references to the manuscript:**

**1. Reference** **4.** Teklehaimanot DA, Mekuria AD, Dadi AF, Derseh BT. Precancerous lesion determinants in women attending cervical cancer screening at public health facilities in North Shoa Zone, Amhara, Ethiopia: an unmatched case-control study. BMC Women's Health. 2024;24(1):271.

**2. Reference 5**. Tesfaye E, Kumbi B, Mandefro B, Hemba Y, Prajapati KK, Singh SC, et al. Prevalence of human papillomavirus infection and associated factors among women attending cervical cancer screening in the setting of Addis Ababa, Ethiopia. Scientific Reports. 2024;14(1):4053.

**3. Reference 6**. Temesgen MM, Alemu T, Shiferaw B, Legesse S, Zeru T, Haile M, et al. Prevalence of oncogenic human papillomavirus (HPV 16/18) infection, cervical lesions, and their associated factors among women aged 21–49 years in Amhara region, Northern Ethiopia. 2021;16(3):e0248949.

**4. Reference 11**. Shapiro GKJCO. HPV vaccination: an underused strategy for the prevention of cancer. 2022;29(5):3780-92.

**5. Reference** **16.** Enyew EB, Kasaye MD, Kebede SD, Feyisa MS, Serbessa NG, Tebeje TM, et al. Human Papillomavirus Vaccination uptake and associated factors among school girls aged between 9–14 years in Ethiopia: Performance Monitoring for Action (PMA-ET) 2023, multilevel analysis. 2025;20(6):e0325557.

**6. Reference** **19.** Rincon NL, McDowell KR, Weatherspoon D, Ritchwood TD, Rocke DJ, Adjei Boakye E, et al. Racial and ethnic disparities in human papillomavirus (HPV) vaccine uptake among United States adults, aged 27–45 years. 2024;20(1):2313249.

**7. Reference** **24**. Aguboshim FCJWJoAR, Reviews. Adequacy of sample size in a qualitative case study and the dilemma of data saturation: A narrative review. 2021;10(3):180-7.

**8. Reference** **25**. Naeem M, Ozuem W, Howell K, Ranfagni SJIJoQM. Demystification and actualisation of data saturation in qualitative research through thematic analysis. 2024;23:16094069241229777.

**Response to reviewer #1 comments**

**Comment 1**: Your data statement still reads “Yes - all data are fully available without restriction”. Then, on page 16, line 445, you state: “Data availability: All important data were available with a reasonable request from the corresponding author.” These are contradictory. Please update

**Response 1:** Thanks for your comment. The sentence is corrected to “All important data were available within the manuscript.” On page 17, Line 463

**Comment 2:** I’m not sure that you did this. The text now reads: “Cervical cancer (CC) is the fourth most frequently diagnosed and fourth most common cause of 64 cancer-related mortality, accounting for 604,127 new cases and 341,831 deaths globally in 2020.”
ν The source you cite states: “Cervical cancer is the fourth most frequently diagnosed cancer and the fourth leading cause of cancer death in women, with an estimated 604,000 new cases and 342,000 deaths worldwide in 2020 (Table 1, Fig.

The words are very similar, but the meaning is very different, as this is in women. What you have written is incorrect.

**Response 2:** Thank you for your feedback. The sentence is updated accordingly on page 3, line 63.

**Comment 3:** ----Update: ---
There are issues with the quality of the English grammar. ‘Purposive sampling’ is not correct English. ‘Conveniently recruited’ is not correct English. You could state, e.g., ‘we used purposive sampling’ or ‘purposive sampling was used’. Equally, ‘We used convenience sampling’ or ‘convenience sampling was used’.

**Response 3:** Thank you for your feedback. It was updated on page 5, line 136 and line 138, and page 7, line 203.

**Comment 4:** ----Update: ---
ν The text now states: “Parents' interviews were categorized into two major themes based on the objectives of the study.” So were some interviews in just one category? Were some in both? Do you mean coded? Categorization implies that participants were not in both categories.

**Response 4:** Thank you for your valuable comment. We have updated it on Page 8, Line 217

**Response to reviewer #2 comments**

ABSTRACT
**Comment 1:** Lines 49 to 50- Perceived facilitators and barriers did not emerge as themes because the authors already stated those as their objective. Alternative wording could be, “findings were split between two broad themes…”

**Response 1:** Thank you for your comment. The Sentence was changed to “Findings were split between two major themes: perceived facilitators and barriers to human papillomavirus vaccine acceptance.” on page 2, line 49 – 50

**Comment 2:** Line 66- remove “being” in “being located”

**Response 2:** Thank you for your comment. “Being” is removed from “being located” on page 3, line 66.

**Comment 3:** Line 71- mention the well-known risk factors

**Response 3:** Thank you for your constructive comment. The well-known risk factors are mentioned on Page 3, lines 72-74.

**Comment 4:** Line 80- Cite the first sentence.

**Response 4**: Thank you for your comment. The first sentence is cited appropriately on page 4, line 83.

**Comment 5:** Line 97- “Recent studies show that…”-citation should be more than 1 based on this statement.

**Response 5:** Thank you for your comment. More than one study is cited based on the suggestion on page 4, lines 101 and 102.

**Comment 6**: Lines 106 to 107- Stating there are no studies is a pretty strong statement and subjective. A better alternative could be to state something along the lines of “to the nest of our knowledge, no studies have…”

**Response 6:** Thank you for your comment. Updated according to the comment on Page 4, line 110.
METHODS
**Comment 7:** Include a conceptual foundation or paradigm on which your study is based - such as critical realist, constructivist, grounded theory, etc.

**Response 7:** Thank you for your insightful recommendation. We agree that a conceptual foundation can give qualitative research a crucial perspective. In this instance, we have purposefully chosen a basic descriptive approach, concentrating on identifying attitudes, enablers, and obstacles to HPV vaccine use among rural parents in the Alle district. We see this descriptive design and content analysis as a first step to lead to deeper exploration in the future using other qualitative methodologies.

Please let us know if further clarification would be helpful.

**Comment 8:** Lines 137 to 138-include citation

**Response 8:** Thank you for your comment. Citation added to it on page 5, lines 142 &143.
RESULTS
**Comment 9:** Line 287- Change to “Other participants also expressed”

**Response 9:** Thank you for your comment. “Another participant also expressed” changed to “Other participants also expressed” on Page 11, line 292, according to the comment.
**Comment 10:** Sub-themes on misconceptions that have quotations about fertility should be added to “fear of side-effects” or vice versa, depending on the entire context of participants’ accounts. It is a bit unclear how the quotations on fertility differ between these 2 sub-themes.

**Response 10:** Thank you for the comment. Concerning the possible overlap between the sub-themes "Misconceptions" and "Fear of Side Effects," especially about participant quotes about fertility, we value your remark. It is sometimes ambiguous to separate themes, but, in our study, under the misconceptions sub-theme, we have identified comments about fertility that contain medical misinformation such as "the vaccine makes girls infertile" and we have grouped quotes that convey general worry or fear of unidentified side effects under fear of side effects, and have not included those here that contain false information.

DISCUSSION
**Comment 11:** The authors provided a summary of the results in the discussion and did not do much of a discussion. Studies cited were not expanded on how they differed or aligned with the current study. Implications of the findings, thoughts around why the results were what they were, should be discussed, backed by past literature (whether for or against). Of value could be other studies in rural areas that have examined HPV vaccination-whether quantitative or qualitative, and studies abroad, e.g. US, can also be cited.

**Response 11:** Thanks for your comment. We value your comment, and we have updated parts of the discussions again.
**Comment 12:** Lines 400, 403 to 404- include a summary of the studies mentioned.

**Response 12:** Thank you for your comment. The summary of the studies is added on page 15, lines 409-4012, and lines 415-417.

CONCLUSION
**Comment 13:** The authors should also mention something about leveraging parents’ perceptions about the benefits. Pushing that messaging forward could also be helpful, as those were found to be directly correlated with vaccine acceptance.

**Response 13:** Thank you for your suggestion. Revised based on the comment on page 16, lines 448-452.
