# Supplementary material for: Perceived facilitators and barriers against human papillomavirus vaccine among rural parents with eligible daughters in the Alle district, Southern Ethiopia
Source: PLoS One. 2026 Jul 22;21(7):e0354372. doi: 10.1371/journal.pone.0354372 (PMC13390824; doi:10.1371/journal.pone.0354372)
Supplement: Peer review history

# Peer review: Decision Letter 0

**DOI:** 10.1371/journal.pone.0354372.r001

**Authors:** Nontuthuzelo Somdyala

18 Mar 2025

PONE-D-24-58812

Perceived facilitators and barriers against human papillomavirus vaccine among rural parents with eligible daughters in the Alle district, Southern Ethiopia.

PLOS ONE

Dear Dr. Zenebe,

Thank you for submitting your manuscript to PLOS ONE. After careful consideration, we feel that it has merit but does not fully meet PLOS ONE’s publication criteria as it currently stands. Therefore, we invite you to submit a revised version of the manuscript that addresses the points raised during the review process.

==============================

**Thank you for sharing your study to the PlosOne Journal for evaluation after which be considered for publication. Your study is valuable for the continent where cancer is increasing but resources not enough to address this increasing problem.**

==============================

Please submit your revised manuscript by May 02 2025 11:59PM. If you will need more time than this to complete your revisions, please reply to this message or contact the journal office at plosone@plos.org. When you're ready to submit your revision, log on to https://www.editorialmanager.com/pone/ and select the 'Submissions Needing Revision' folder to locate your manuscript file.

Please include the following items when submitting your revised manuscript:

- A rebuttal letter that responds to each point raised by the academic editor and reviewer(s). You should upload this letter as a separate file labeled 'Response to Reviewers'.
- A marked-up copy of your manuscript that highlights changes made to the original version. You should upload this as a separate file labeled 'Revised Manuscript with Track Changes'.
- An unmarked version of your revised paper without tracked changes. You should upload this as a separate file labeled 'Manuscript'.

-->If you would like to make changes to your financial disclosure, please include your updated statement in your cover letter. Guidelines for resubmitting your figure files are available below the reviewer comments at the end of this letter.

If applicable, we recommend that you deposit your laboratory protocols in protocols.io to enhance the reproducibility of your results. Protocols.io assigns your protocol its own identifier (DOI) so that it can be cited independently in the future. For instructions see: https://journals.plos.org/plosone/s/submission-guidelines#loc-laboratory-protocols. Additionally, PLOS ONE offers an option for publishing peer-reviewed Lab Protocol articles, which describe protocols hosted on protocols.io. Read more information on sharing protocols at https://plos.org/protocols?utm_medium=editorial-email&utm_source=authorletters&utm_campaign=protocols.

We look forward to receiving your revised manuscript.

Kind regards,

Nontuthuzelo Iris Muriel Somdyala, Ph.D

Academic Editor

PLOS ONE

Journal requirements:

When submitting your revision, we need you to address these additional requirements.

1. Please ensure that your manuscript meets PLOS ONE's style requirements, including those for file naming. The PLOS ONE style templates can be found at

https://journals.plos.org/plosone/s/file?id=wjVg/PLOSOne_formatting_sample_main_body.pdf and

https://journals.plos.org/plosone/s/file?id=ba62/PLOSOne_formatting_sample_title_authors_affiliations.pdf.

2. In the online submission form, you indicated that [All important data were available with a reasonable request from the corresponding author.].

All PLOS journals now require all data underlying the findings described in their manuscript to be freely available to other researchers, either 1. In a public repository, 2. Within the manuscript itself, or 3. Uploaded as supplementary information.

This policy applies to all data except where public deposition would breach compliance with the protocol approved by your research ethics board. If your data cannot be made publicly available for ethical or legal reasons (e.g., public availability would compromise patient privacy), please explain your reasons on resubmission and your exemption request will be escalated for approval.

3. Your ethics statement should only appear in the Methods section of your manuscript. If your ethics statement is written in any section besides the Methods, please delete it from any other section.

4. Please review your reference list to ensure that it is complete and correct. If you have cited papers that have been retracted, please include the rationale for doing so in the manuscript text, or remove these references and replace them with relevant current references. Any changes to the reference list should be mentioned in the rebuttal letter that accompanies your revised manuscript. If you need to cite a retracted article, indicate the article’s retracted status in the References list and also include a citation and full reference for the retraction notice.

[Note: HTML markup is below. Please do not edit.]

Reviewers' comments:

Reviewer's Responses to Questions

**Comments to the Author**

1. Is the manuscript technically sound, and do the data support the conclusions?

The manuscript must describe a technically sound piece of scientific research with data that supports the conclusions. Experiments must have been conducted rigorously, with appropriate controls, replication, and sample sizes. The conclusions must be drawn appropriately based on the data presented.

Reviewer #1: Yes

**********

2. Has the statistical analysis been performed appropriately and rigorously?

Reviewer #1: N/A

**********

3. Have the authors made all data underlying the findings in their manuscript fully available?

The PLOS Data policy requires authors to make all data underlying the findings described in their manuscript fully available without restriction, with rare exception (please refer to the Data Availability Statement in the manuscript PDF file). The data should be provided as part of the manuscript or its supporting information, or deposited to a public repository. For example, in addition to summary statistics, the data points behind means, medians and variance measures should be available. If there are restrictions on publicly sharing data—e.g. participant privacy or use of data from a third party—those must be specified.

Reviewer #1: Yes

**********

4. Is the manuscript presented in an intelligible fashion and written in standard English?

PLOS ONE does not copyedit accepted manuscripts, so the language in submitted articles must be clear, correct, and unambiguous. Any typographical or grammatical errors should be corrected at revision, so please note any specific errors here.

Reviewer #1: Yes

**********

5. Review Comments to the Author

Please use the space provided to explain your answers to the questions above. You may also include additional comments for the author, including concerns about dual publication, research ethics, or publication ethics. (Please upload your review as an attachment if it exceeds 20,000 characters)

Reviewer #1: Overall, I think the research was well executed and is about an extremely important topic. It’s very exciting to read about research from Southern Ethiopia and the manuscript will be of interest to an international audience. I have only minor comments.

Data availability statement:

I think more clarity is needed here. You state “Yes - all data are fully available without restriction” – would this be anonymised or de-identified data? Are all data underlying the findings in their manuscript already available in the manuscript?

Abstract: the gender ratio of the participants and the religions of the participants would be of interest to the reader here.

Page 3, line 52: “Cervical cancer (CC) is the ninth most common and also the ninth most frequent cause of new 63 deaths among overall cancer cases. (1)” Is it the ninth most common incident type of cancer and the ninth most common cause of death by cancer? They way this is written, it could be the ninth most common cause of death by cancer and also the ninth most frequent cause of death by cancer (and these are the same). Also, as cervical cancer doesn’t affect men, is it better to delete this sentence and open with the second sentence (which is about a female only sample)? The rest of this paragraph contains really important information.

Page 3, line 71 “these rates are expected to intensify and double by 2040.” – can you let the reader know why?

Page 5, line 120: “A qualitative study was used.” Can you provide more information – e.g. thematic analysis.

Throughout the manuscript, you refer to “conveniently sampled” – this is called ‘convenience sampling’ – e.g. convenience sampling was used. Can you let the reader know how this worked? E.g. flyers, leaflets, the suggestion from health extension workers? Were participants offered a reward for taking part in the study?

Page 5, line 134: “groups that considered homogeneity in characteristics and educational level” – so did you try to get all the groups to be as similar as possible to one-another? Why? Often in qualitative research we aim for heterogeneity (to have diversity of opinion).

Page 6, line 160: “A semi-structured FGD guide was used as a guide during moderation”: I’m not sure what this means – ‘a guide was used as a guide’ – maybe this text can be simplified. What is moderation in this context?

Page 6, line 170: “The 170 data was analyzed” – the data were analysed.

Page 6, line 170: “Major themes were derived based on the objective of the study. However, subthemes were derived from the text itself through repeated reading.” – so it seems that you used a mixture of inductive and deductive coding. If you agree, it would be helpful for readers to know this and you could put it at the start of the study design section, for example.

Page 7, line 197: “purposely selected for the in-depth interview based on their data taken from local health extension workers.” Can you tell us about the criteria you used to determine the purposive sampling – e.g. vaccine refusal? Strong beliefs? Verbally skilled? How many criteria were used?

Page 7, line 201: “Regarding the level of education, 43 (63.2%) parents had completed the primary level of education, and 25 parents had a secondary level of education and above” For international readers, it would be useful to have clarity about the age at which these levels of education are typically achieved.

Page 8, line 204: “Two categories of major themes were identified from the parents' interviews. These are the facilitators and barriers to accepting the HPV vaccine.” This statement contradicts earlier information that told us that the major themes were determined by the objectives.

Page 15, line 422: “Misconceptions, lack of trust, and different barriers resulted in extremely low parental understanding and acceptance of the HPV vaccine”. This seems to contrast with page 9, line 247: “Most parents in the FGDs and IDIs said that they believe in the information from health experts 248 and recommend that information be provided to the parents. Parents expressed that they believe 249 in the recommendations of the health experts and are open to hearing from them about the HPV 250 vaccines since they have no other trusted information.” How would you characterise extremely low parental acceptance? What were the vaccine acceptance rates? Is that information available?

Page 15, line 424 – do you think addressing misinformation should be a key recommendation from the paper? For example, the concerns about outside interference (the influences of foreign policies), the vaccine being unnecessary, reducing fertility, and being used to control the birth rate, confusion about HPV and HIV, uncertainty about what cancer is – do these rumours need targeting head on? Are there ways of preparing health workers to have these conversations (in addition to communicating about the benefits)?

Overall:

It would be nice for the reader to know more about the decision making process between the parents regarding vaccine acceptance. Did you collect data about whether the mother or father tends to decide? How do they reach the decision? Did you interview any mothers and fathers of the same child together? This supports readers to understand the context and maybe understand whether this context is similar to the context in which they are working.

In summary, thank you for sharing this important work with the international community.

**********

6. PLOS authors have the option to publish the peer review history of their article (what does this mean?). If published, this will include your full peer review and any attached files.

If you choose “no”, your identity will remain anonymous but your review may still be made public.

**Do you want your identity to be public for this peer review?**  For information about this choice, including consent withdrawal, please see our Privacy Policy.

Reviewer #1: No

**********

[NOTE: If reviewer comments were submitted as an attachment file, they will be attached to this email and accessible via the submission site. Please log into your account, locate the manuscript record, and check for the action link "View Attachments". If this link does not appear, there are no attachment files.]

While revising your submission, please upload your figure files to the Preflight Analysis and Conversion Engine (PACE) digital diagnostic tool, https://pacev2.apexcovantage.com/. PACE helps ensure that figures meet PLOS requirements. To use PACE, you must first register as a user. Registration is free. Then, login and navigate to the UPLOAD tab, where you will find detailed instructions on how to use the tool. If you encounter any issues or have any questions when using PACE, please email PLOS at figures@plos.org. Please note that Supporting Information files do not need this step.

# Author comment: Author response to Decision Letter 1

**DOI:** 10.1371/journal.pone.0354372.r002

25 Apr 2025

Dear Academic Editor,

We are deeply grateful for the invaluable comments and recommendations provided by you and the reviewers. Your insights have significantly improved the quality and relevance of our manuscript, and we have carefully addressed each point raised. We have highlighted the changes made in the revised manuscript and attached a point-by-point response to the reviewers' comments as requested. Your expert guidance and dedication throughout the review process have been instrumental in enhancing the overall rigor of our work.

Thank you for your thoughtful review and support.

Warm regards,

Selemaye Zenebe, corresponding author

Arba Minch University, selemayez@gmail.com

Response to reviewer #1 comments

Comment 1: Overall, I think the research was well executed and is about an extremely important topic. It’s very exciting to read about research from Southern Ethiopia and the manuscript will be of interest to an international audience. I have only minor comments.

Response 1: Thank you for your insightful feedback. We appreciate your acknowledgment of the relevance of our work.

Comment 2: Data availability statement: I think more clarity is needed here. You state “Yes - all data are fully available without restriction” – would this be anonymized or de-identified data? Are all data underlying the findings in their manuscript already available in the manuscript?

Response 2: Thank you for your feedback. Deidentified data were used. All the underlying data are available in the manuscript.

Comment 3: Abstract: the gender ratio of the participants and the religions of the participants would be of interest to the reader here.

Response 3: Thank you for your valuable suggestion. We have added the gender ratio and religion of the participants in the abstract on page 2, line 48.

Comment 4: Page 3, line 52: “Cervical cancer (CC) is the ninth most common and also the ninth most frequent cause of new deaths among overall cancer cases. (1)” Is it the ninth most common incident type of cancer and the ninth most common cause of death by cancer? The way this is written, it could be the ninth most common cause of death by cancer and also the ninth most frequent cause of death by cancer (and these are the same). Also, as cervical cancer doesn’t affect men, is it better to delete this sentence and open with the second sentence (which is about a female-only sample)? The rest of this paragraph contains really important information.

Response 4: Thank you for your feedback. The first sentence was deleted and the paragraph opened with the second sentence in On Page 3, line 63.

Comment 5: Page 3, line 71 “These rates are expected to intensify and double by 2040.” – can you let the reader know why?

Response 5: Thank you for your feedback. The reasons why the rate will intensify and double by 2040 are updated in the revised manuscript on Page 3, line 70.

Comment 6: Page 5, line 120: “A qualitative study was used.” Can you provide more information – e.g. thematic analysis?

Response 6: Thank you for your feedback. We added information about the analytical method on Page 5, line 120.

Comment 7: Throughout the manuscript, you refer to “conveniently sampled” – this is called ‘convenience sampling’ – e.g. convenience sampling was used. Can you let the reader know how this worked? E.g. flyers, leaflets, the suggestions from health extension workers? Were participants offered a reward for taking part in the study?

Response 7: Thank you for your feedback. The researchers used convenience sampling with subjects referred by health extension workers and the participants were not paid to participate explained on Page 5, line 133, and page 5, line 139.

Comment 8: Page 5, line 134: “groups that considered homogeneity in characteristics and educational level” – so did you try to get all the groups to be as similar as possible to one another? Why? Often in qualitative research, we aim for heterogeneity (to have diversity of opinion).

Response 8: Thank you for your feedback. Page 5, line 134: For FGD the participants were assigned to groups that were considered homogeneous in sex and heterogeneous in characteristics and educational level. The groups were organized into two male groups and three female groups (expressed on Page 5, lines 134&135) with heterogeneity in other socio-demographic aspects. This was because of the cultural views of the society and the women could not freely express their ideas in front of the male parents.

Comment 9: Page 6, line 160: “A semi-structured FGD guide was used as a guide during moderation”: I’m not sure what this means – ‘a guide was used as a guide’ – maybe this text can be simplified. What is moderation in this context?

Response 9: Thank you for the feedback. The sentence was simplified to: Focus Group Discussions (FGDs) Guide: A semi-structured FGD guide was used to direct the conversation after adaptation from existing literature on page 6, line 162 in the revised manuscript.

Comment 10: Page 6, line 170: “The data was analyzed” – the data were analyzed.

Response 10: Thank you for the feedback. Page 6, line 171 on the revised manuscript: “Was” was replaced by “were”.

Comment 11: Page 6, line 170: “Major themes were derived based on the objective of the study. However, subthemes were derived from the text itself through repeated reading.” – so it seems that you used a mixture of inductive and deductive coding. If you agree, it would be helpful for readers to know this and you could put it at the start of the study design section, for example.

Response 11: Thank you for the feedback. Yes in our study we used both inductive and deductive coding to include core components that don’t fit with the initially prepared framework of the major themes. We added it to the revised manuscript on the method section page 7, line 172.

Comment 12: Page 7, line 197: “purposely selected for the in-depth interview based on their data taken from local health extension workers.” Can you tell us about the criteria you used to determine the purposive sampling – e.g. vaccine refusal? Strong beliefs? Verbally skilled? How many criteria were used?

Response 12: Thank you for the feedback. Page 7, line 197: “The participants were purposely selected for the in-depth interview based on their data taken from local health extension workers. The criteria for selection were addressed on page 7, lines 200-203 of the revised manuscript.

Comment 13: Page 7, line 201: “Regarding the level of education, 43 (63.2%) parents had completed the primary level of education, and 25 parents had a secondary level of education and above” For international readers, it would be useful to have clarity about the age at which these levels of education are typically achieved.

Response 13: Thank you for your feedback. We think it is difficult to put a specific age at which the education level was achieved. Since it may be achieved at different ages according to access to education, the time someone started school, school drop rates, and the like. Different studies in our nation reported like ours to specify their education level as primary(grade 1-8), secondary education (grade 9-12), and above means college certificates up to university degrees. We added supportive information on page 8, lines 207 – 209 of the revised manuscript.

Comment 14: Page 8, line 204: “Two categories of major themes were identified from the parents' interviews. These are the facilitators and barriers to accepting the HPV vaccine.” This statement contradicts earlier information that told us that the major themes were determined by the objectives.

Response 14: Thank you for your feedback. Page 8, line 204: The two major themes were identified based on the objectives but the subthemes for each major theme were identified based on the parent's interviews. These two methods were used to include core components that don’t fit with the initially prepared framework of the major themes from the objective. So we corrected it accordingly and found it on page 8, line 212 of the revised manuscript.

Comment 15: Page 15, line 422: “Misconceptions, lack of trust, and different barriers resulted in extremely low parental understanding and acceptance of the HPV vaccine”. This seems to contrast with page 9, line 247: “Most parents in the FGDs and IDIs said that they believe in the information from health experts 248 and recommend that information be provided to the parents. Parents expressed that they believe 249 in the recommendations of the health experts and are open to hearing from them about the HPV 250 vaccines since they have no other trusted information.” How would you characterize extremely low parental acceptance? What were the vaccine acceptance rates? Is that information available?

Response 15: Thank you for your feedback. We have updated it and also we have added justification here for the reviewer. Page 15, line 422: it may seem to contrast with page 9, line 247: but the idea from page 9 line 247 refers to the fact that the parents trust the recommendations and information from health experts but they are also exposed to other sources of information including misconceptions, lack of trust in the HPV vaccine and others treatments which resulted in an unwillingness to vaccinate their daughter’s. There was no information available on the actual rate of vaccine acceptance.

Comment 16: Page 15, line 424 – do you think addressing misinformation should be a key recommendation from the paper? For example, the concerns about outside interference (the influences of foreign policies), the vaccine is unnecessary, reducing fertility, and being used to control the birth rate, confusion about HPV and HIV, and uncertainty about what cancer is – do these rumors need targeting head on? Are there ways of preparing health workers to have these conversations (in addition to communicating about the benefits)?

Response 16: Thank you for your feedback. Page 15, line 424: We agree with your observation that there is a need to dispel these misconceptions. Addressed on page 16, line 430 in the revised manuscript.

Attachment Submitted filename: Response to Reviewers.docx

# Peer review: Decision Letter 1

**DOI:** 10.1371/journal.pone.0354372.r003

**Authors:** Kehinde Kanmodi

23 Jul 2025

PONE-D-24-58812R1

Perceived facilitators and barriers against human papillomavirus vaccine among rural parents with eligible daughters in the Alle district, Southern Ethiopia.

PLOS ONE

Dear Dr. Zenebe,

Thank you for submitting your manuscript to PLOS ONE. After careful consideration, we feel that it has merit but does not fully meet PLOS ONE’s publication criteria as it currently stands. Therefore, we invite you to submit a revised version of the manuscript that addresses the points raised during the review process.

Please submit your revised manuscript by Sep 06 2025 11:59PM. If you will need more time than this to complete your revisions, please reply to this message or contact the journal office at plosone@plos.org. When you're ready to submit your revision, log on to https://www.editorialmanager.com/pone/ and select the 'Submissions Needing Revision' folder to locate your manuscript file.

Please include the following items when submitting your revised manuscript:

- A rebuttal letter that responds to each point raised by the academic editor and reviewer(s). You should upload this letter as a separate file labeled 'Response to Reviewers'.
- A marked-up copy of your manuscript that highlights changes made to the original version. You should upload this as a separate file labeled 'Revised Manuscript with Track Changes'.
- An unmarked version of your revised paper without tracked changes. You should upload this as a separate file labeled 'Manuscript'.

-->If you would like to make changes to your financial disclosure, please include your updated statement in your cover letter. Guidelines for resubmitting your figure files are available below the reviewer comments at the end of this letter.

If applicable, we recommend that you deposit your laboratory protocols in protocols.io to enhance the reproducibility of your results. Protocols.io assigns your protocol its own identifier (DOI) so that it can be cited independently in the future. For instructions see: https://journals.plos.org/plosone/s/submission-guidelines#loc-laboratory-protocols. Additionally, PLOS ONE offers an option for publishing peer-reviewed Lab Protocol articles, which describe protocols hosted on protocols.io. Read more information on sharing protocols at https://plos.org/protocols?utm_medium=editorial-email&utm_source=authorletters&utm_campaign=protocols.

We look forward to receiving your revised manuscript.

Kind regards,

Kehinde Kazeem Kanmodi, BDS, MPH

Academic Editor

PLOS ONE

Journal Requirements:

If the reviewer comments include a recommendation to cite specific previously published works, please review and evaluate these publications to determine whether they are relevant and should be cited. There is no requirement to cite these works unless the editor has indicated otherwise.

Please review your reference list to ensure that it is complete and correct. If you have cited papers that have been retracted, please include the rationale for doing so in the manuscript text, or remove these references and replace them with relevant current references. Any changes to the reference list should be mentioned in the rebuttal letter that accompanies your revised manuscript. If you need to cite a retracted article, indicate the article’s retracted status in the References list and also include a citation and full reference for the retraction notice.

[Note: HTML markup is below. Please do not edit.]

Reviewers' comments:

Reviewer's Responses to Questions

**Comments to the Author**

1. If the authors have adequately addressed your comments raised in a previous round of review and you feel that this manuscript is now acceptable for publication, you may indicate that here to bypass the “Comments to the Author” section, enter your conflict of interest statement in the “Confidential to Editor” section, and submit your "Accept" recommendation.

Reviewer #1: (No Response)

Reviewer #2: (No Response)

**********

2. Is the manuscript technically sound, and do the data support the conclusions?

The manuscript must describe a technically sound piece of scientific research with data that supports the conclusions. Experiments must have been conducted rigorously, with appropriate controls, replication, and sample sizes. The conclusions must be drawn appropriately based on the data presented.

Reviewer #1: Yes

Reviewer #2: Yes

**********

3. Has the statistical analysis been performed appropriately and rigorously?

Reviewer #1: N/A

Reviewer #2: Yes

**********

4. Have the authors made all data underlying the findings in their manuscript fully available?

The PLOS Data policy requires authors to make all data underlying the findings described in their manuscript fully available without restriction, with rare exception (please refer to the Data Availability Statement in the manuscript PDF file). The data should be provided as part of the manuscript or its supporting information, or deposited to a public repository. For example, in addition to summary statistics, the data points behind means, medians and variance measures should be available. If there are restrictions on publicly sharing data—e.g. participant privacy or use of data from a third party—those must be specified.

Reviewer #1: Yes

Reviewer #2: Yes

**********

5. Is the manuscript presented in an intelligible fashion and written in standard English?

PLOS ONE does not copyedit accepted manuscripts, so the language in submitted articles must be clear, correct, and unambiguous. Any typographical or grammatical errors should be corrected at revision, so please note any specific errors here.

Reviewer #1: Yes

Reviewer #2: Yes

**********

6. Review Comments to the Author

Please use the space provided to explain your answers to the questions above. You may also include additional comments for the author, including concerns about dual publication, research ethics, or publication ethics. (Please upload your review as an attachment if it exceeds 20,000 characters)

Reviewer #1: There are still some outstanding comments from the first peer review to address, copied below with an update.

Comment 2: Data availability statement: I think more clarity is needed here. You state “Yes - all data are fully available without restriction” – would this be anonymized or de-identified data? Are all data underlying the findings in their manuscript already available in the manuscript?

Response 2: Thank you for your feedback. Deidentified data were used. All the underlying data are available in the manuscript.

----Update: ---

• Your data statement still reads “Yes - all data are fully available without restriction”. Then on page 16, line 445, you state:

• “Data availability: All important data were available with a reasonable request from the corresponding author.”

• These are contradictory. Please update.

Comment 4: Page 3, line 52: “Cervical cancer (CC) is the ninth most common and also the ninth most frequent cause of new deaths among overall cancer cases. (1)” Is it the ninth most common incident type of cancer and the ninth most common cause of death by cancer? The way this is written, it could be the ninth most common cause of death by cancer and also the ninth most frequent cause of death by cancer (and these are the same). Also, as cervical cancer doesn’t affect men, is it better to delete this sentence and open with the second sentence (which is about a female-only sample)? The rest of this paragraph contains really important information.

Response 4: Thank you for your feedback. The first sentence was deleted and the paragraph opened with the second sentence in On Page 3, line 63.

----Update: ---

• I’m not sure that you did this. The text now reads: “Cervical cancer (CC) is the fourth most frequently diagnosed and fourth most common cause of 64 cancer-related mortality accounting for 604,127 new cases and 341,831 deaths globally in 2020.”

• The source you cite states: “Cervical cancer is the fourth most frequently diagnosed cancer and the fourth leading cause of cancer death in women, with an estimated 604,000 new cases and 342,000 deaths worldwide in 2020 (Table 1, Fig. 4). ”

• The words are very similar, but the meaning is very different as this is in women. What you have written is incorrect.

Comment 7: Throughout the manuscript, you refer to “conveniently sampled” – this is called ‘convenience sampling’ – e.g. convenience sampling was used. Can you let the reader know how this worked? E.g. flyers, leaflets, the suggestions from health extension workers? Were participants offered a reward for taking part in the study?

Response 7: Thank you for your feedback. The researchers used convenience sampling with subjects referred by health extension workers and the participants were not paid to participate explained on Page 5, line 133, and page 5, line 139.

----Update: ---

• There are issues with the quality of the English grammar. ‘Purposive samplings’ is not correct English. ‘Conveniently recruited’ is not correct English. You could state, e.g. ‘we used purposive sampling’ or ‘purposive sampling was used’. Equally, ‘We used convenience sampling’ or ‘convenience sampling was used’.

Comment 14: Page 8, line 204: “Two categories of major themes were identified from the parents' interviews. These are the facilitators and barriers to accepting the HPV vaccine.” This statement contradicts earlier information that told us that the major themes were determined by the objectives.

Response 14: Thank you for your feedback. Page 8, line 204: The two major themes were identified based on the objectives but the subthemes for each major theme were identified based on the parent's interviews. These two methods were used to include core components that don’t fit with the initially prepared framework of the major themes from the objective. So we corrected it accordingly and found it on page 8, line 212 of the revised manuscript.

----Update: ---

• The text now states: “Parents' interviews were categorized into two major themes based on the objectives of the study.” So were some interviews in just one category? Were some in both? Do you mean coded? Categorised implies that participants were not in both categories.

Reviewer #2: REVIEWER COMMENTS

Study is impactful and addresses need in an underserved area. Participants’ perspectives were quite rich and should add to the body of knowledge in HPV vaccination in sub-Saharan Africa. Authors were responsive to previous reviewers’ comments. I provided some suggestions for more revision, mostly minor. However, I feel the theoretical underpinning for this research and a more comprehensive discussion should be done.

ABSTRACT

Lines 49 to 50- Perceived facilitators and barriers did not emerge as themes because the authors already stated those as their objective. Alternative wording could be, “findings were split between two broad themes…”

INTRODUCTION

Line 66- remove “being” in “being located”

Line 71- mention the well-known risk factors

Line 80- Cite the first sentence

Line 97- “Recent studies show that…”-citation should be more than 1 based on this statement.

Lines 106 to 107- Stating there are no studies is a pretty strong statement and subjective. A better alternative could be to state something along the lines of “to the nest of our knowledge, no studies have…”.

METHODS

Include a conceptual foundation or paradigm on which your study is based on- such as critical realist, constructivist, grounded theory, etc.

Lines 137 to 138-include citation

RESULTS

Line 287- Change to “Other participants also expressed”

Sub-themes on Misconceptions that have quotations about fertility should be added to “fear of side-effects” or vice versa depending on the entire context of participants’ accounts. It is a bit unclear how the quotations on fertility differ between these 2 sub themes.

DISCUSSION

The authors provided a summary of the results in the discussion and did not do so much of a discussion. Studies cited were not expanded on how they differed or aligned with the current study. Implications of the findings, thoughts around why the results were what they were should be discussed, backed by past literature (whether for or against). Of value could be other studies in rural areas that have examined HPV vaccination-whether quantitative or qualitative and studies abroad eg US can also be cited.

Lines 400, 403 to 404- include a summary of the studies mentioned.

CONCLUSION

The authors should also mention something around leveraging parents’ perceptions about the benefits. Pushing that messaging forward could also be helpful as those were found to be directly correlated with vaccine acceptance.

**********

7. PLOS authors have the option to publish the peer review history of their article (what does this mean?). If published, this will include your full peer review and any attached files.

If you choose “no”, your identity will remain anonymous but your review may still be made public.

**Do you want your identity to be public for this peer review?**  For information about this choice, including consent withdrawal, please see our Privacy Policy.

Reviewer #1: No

Reviewer #2: No

**********

[NOTE: If reviewer comments were submitted as an attachment file, they will be attached to this email and accessible via the submission site. Please log into your account, locate the manuscript record, and check for the action link "View Attachments". If this link does not appear, there are no attachment files.]

While revising your submission, please upload your figure files to the Preflight Analysis and Conversion Engine (PACE) digital diagnostic tool, https://pacev2.apexcovantage.com/. PACE helps ensure that figures meet PLOS requirements. To use PACE, you must first register as a user. Registration is free. Then, login and navigate to the UPLOAD tab, where you will find detailed instructions on how to use the tool. If you encounter any issues or have any questions when using PACE, please email PLOS at figures@plos.org. Please note that Supporting Information files do not need this step.

# Author comment: Author response to Decision Letter 2

**DOI:** 10.1371/journal.pone.0354372.r004

30 Aug 2025

Dear Academic Editor,

We are deeply grateful for the invaluable comments and recommendations provided by you and the reviewers once again. Your insights have significantly improved the quality and relevance of our manuscript, and we have carefully addressed each point raised. We have highlighted the changes made in the revised manuscript and attached a point-by-point response to the reviewers' comments as requested. Your expert guidance and dedication throughout the review process have been instrumental in enhancing the overall rigor of our work.

Thank you for your thoughtful review and support.

Warm regards,

Selemaye Zenebe, corresponding author

Arba Minch University, selemayez@gmail.com

Changes were made to the reference list based on the suggestions and comments from the editor and reviewers

Retracted references that are removed from the manuscript include:

1. Cancer WIAfRo. Cancer Tomorrow: estimated number of cervical cancer incidence and mortality in Ethiopia.

2. Organization. WH. Ethiopia launches Human Papillomavirus Vaccine for 14-year-old Girls, 2018 World Health Organization. 2021

Newly added 8 relevant references to the manuscript:

1. Reference 4. Teklehaimanot DA, Mekuria AD, Dadi AF, Derseh BT. Precancerous lesion determinants in women attending cervical cancer screening at public health facilities in North Shoa Zone, Amhara, Ethiopia: an unmatched case-control study. BMC Women's Health. 2024;24(1):271.

2. Reference 5. Tesfaye E, Kumbi B, Mandefro B, Hemba Y, Prajapati KK, Singh SC, et al. Prevalence of human papillomavirus infection and associated factors among women attending cervical cancer screening in the setting of Addis Ababa, Ethiopia. Scientific Reports. 2024;14(1):4053.

3. Reference 6. Temesgen MM, Alemu T, Shiferaw B, Legesse S, Zeru T, Haile M, et al. Prevalence of oncogenic human papillomavirus (HPV 16/18) infection, cervical lesions, and their associated factors among women aged 21–49 years in Amhara region, Northern Ethiopia. 2021;16(3):e0248949.

4. Reference 11. Shapiro GKJCO. HPV vaccination: an underused strategy for the prevention of cancer. 2022;29(5):3780-92.

5. Reference 16. Enyew EB, Kasaye MD, Kebede SD, Feyisa MS, Serbessa NG, Tebeje TM, et al. Human Papillomavirus Vaccination uptake and associated factors among school girls aged between 9–14 years in Ethiopia: Performance Monitoring for Action (PMA-ET) 2023, multilevel analysis. 2025;20(6):e0325557.

6. Reference 19. Rincon NL, McDowell KR, Weatherspoon D, Ritchwood TD, Rocke DJ, Adjei Boakye E, et al. Racial and ethnic disparities in human papillomavirus (HPV) vaccine uptake among United States adults, aged 27–45 years. 2024;20(1):2313249.

7. Reference 24. Aguboshim FCJWJoAR, Reviews. Adequacy of sample size in a qualitative case study and the dilemma of data saturation: A narrative review. 2021;10(3):180-7.

8. Reference 25. Naeem M, Ozuem W, Howell K, Ranfagni SJIJoQM. Demystification and actualisation of data saturation in qualitative research through thematic analysis. 2024;23:16094069241229777.

Response to reviewer #1 comments

Comment 1: Your data statement still reads “Yes - all data are fully available without restriction”. Then, on page 16, line 445, you state: “Data availability: All important data were available with a reasonable request from the corresponding author.” These are contradictory. Please update

Response 1: Thanks for your comment. The sentence is corrected to “All important data were available within the manuscript.” On page 17, Line 463

Comment 2: I’m not sure that you did this. The text now reads: “Cervical cancer (CC) is the fourth most frequently diagnosed and fourth most common cause of 64 cancer-related mortality, accounting for 604,127 new cases and 341,831 deaths globally in 2020.”

• The source you cite states: “Cervical cancer is the fourth most frequently diagnosed cancer and the fourth leading cause of cancer death in women, with an estimated 604,000 new cases and 342,000 deaths worldwide in 2020 (Table 1, Fig.

The words are very similar, but the meaning is very different, as this is in women. What you have written is incorrect.

Response 2: Thank you for your feedback. The sentence is updated accordingly on page 3, line 63.

Comment 3: ----Update: ---

There are issues with the quality of the English grammar. ‘Purposive sampling’ is not correct English. ‘Conveniently recruited’ is not correct English. You could state, e.g., ‘we used purposive sampling’ or ‘purposive sampling was used’. Equally, ‘We used convenience sampling’ or ‘convenience sampling was used’.

Response 3: Thank you for your feedback. It was updated on page 5, line 136 and line 138, and page 7, line 203.

Comment 4: ----Update: ---

• The text now states: “Parents' interviews were categorized into two major themes based on the objectives of the study.” So were some interviews in just one category? Were some in both? Do you mean coded? Categorization implies that participants were not in both categories.

Response 4: Thank you for your valuable comment. We have updated it on Page 8, Line 217

Response to reviewer #2 comments

ABSTRACT

Comment 1: Lines 49 to 50- Perceived facilitators and barriers did not emerge as themes because the authors already stated those as their objective. Alternative wording could be, “findings were split between two broad themes…”

Response 1: Thank you for your comment. The Sentence was changed to “Findings were split between two major themes: perceived facilitators and barriers to human papillomavirus vaccine acceptance.” on page 2, line 49 – 50

Comment 2: Line 66- remove “being” in “being located”

Response 2: Thank you for your comment. “Being” is removed from “being located” on page 3, line 66.

Comment 3: Line 71- mention the well-known risk factors

Response 3: Thank you for your constructive comment. The well-known risk factors are mentioned on Page 3, lines 72-74.

Comment 4: Line 80- Cite the first sentence.

Response 4: Thank you for your comment. The first sentence is cited appropriately on page 4, line 83.

Comment 5: Line 97- “Recent studies show that…”-citation should be more than 1 based on this statement.

Response 5: Thank you for your comment. More than one study is cited based on the suggestion on page 4, lines 101 and 102.

Comment 6: Lines 106 to 107- Stating there are no studies is a pretty strong statement and subjective. A better alternative could be to state something along the lines of “to the nest of our knowledge, no studies have…”

Response 6: Thank you for your comment. Updated according to the comment on Page 4, line 110.

METHODS

Comment 7: Include a conceptual foundation or paradigm on which your study is based - such as critical realist, constructivist, grounded theory, etc.

Response 7: Thank you for your insightful recommendation. We agree that a conceptual foundation can give qualitative research a crucial perspective. In this instance, we have purposefully chosen a basic descriptive approach, concentrating on identifying attitudes, enablers, and obstacles to HPV vaccine use among rural parents in the Alle district. We see this descriptive design and content analysis as a first step to lead to deeper exploration in the future using other qualitative methodologies.

Please let us know if further clarification would be helpful.

Comment 8: Lines 137 to 138-include citation

Response 8: Thank you for your comment. Citation added to it on page 5, lines 142 &143.

RESULTS

Comment 9: Line 287- Change to “Other participants also expressed”

Response 9: Thank you for your comment. “Another participant also expressed” changed to “Other participants also expressed” on Page 11, line 292, according to the comment.

Comment 10: Sub-themes on misconceptions that have quotations about fertility should be added to “fear of side-effects” or vice versa, depending on the entire context of participants’ accounts. It is a bit unclear how the quotations on fertility differ between these 2 sub-themes.

Response 10: Thank you for the comment. Concerning the possible overlap between the sub-themes "Misconceptions" and "Fear of Side Effects," especially about participant quotes about fertility, we value your remark. It is sometimes ambiguous to separate themes, but, in our study, under the misconceptions sub-theme, we have identified comments about fertility that contain medical misinformation such as "the vaccine makes girls infertile" and we have grouped quotes that convey general worry or fear of unidentified side effects under fear of side effects, and have not included those here that contain false information.

DISCUSSION

Comment 11: The authors provided a summary of the results in the discussion and did not do much of a discussion. Studies cited were not expanded on how they differed or aligned with the current study. Implications of the findings, thoughts around why the results were what they were, should be discussed, backed by past literature (whether for or against). Of value could be other studies in rural areas that have examined HPV vaccination-whether quantitative or qualitative, and studies abroad, e.g. US, can also be cited.

Response 11: Thanks for your comment. We value your comment, and we have updated parts of the discussions again.

Comment 12: Lines 400, 403 to 404- include a summary of the studies mentioned.

Response 12: Thank you for your comment. The summary of the studies is added on page 15, lines 409-4012, and lines 415-417.

CONCLUSION

Comment 13: The authors should also mention something about leveraging parents’ perceptions about the benefits. Pushing that messaging forward could also be helpful, as those were found to be directly correlated with vaccine acceptance.

Response 13: Thank you for your suggestion. Revised based on the comment on page 16, lines 448-452.

Attachment Submitted filename: Response_to_Reviewers_auresp_2.docx

# Editor report: Decision Letter 2

**DOI:** 10.1371/journal.pone.0354372.r005

**Authors:** Ricardo Gurgel

20 Oct 2025

PONE-D-24-58812R2

Perceived facilitators and barriers against human papillomavirus vaccine among rural parents with eligible daughters in the Alle district, Southern Ethiopia.

PLOS ONE

Dear Dr. Zenebe,

Thank you for submitting your manuscript to PLOS ONE. After careful consideration, we feel that it has merit but does not fully meet PLOS ONE’s publication criteria as it currently stands. Therefore, we invite you to submit a revised version of the manuscript that addresses the points raised during the review process.

==================================

Please note that there are some suggestions considered as of substantial importance and need to be faced by the authors.

==================================

Please submit your revised manuscript by Dec 04 2025 11:59PM. If you will need more time than this to complete your revisions, please reply to this message or contact the journal office at plosone@plos.org. When you're ready to submit your revision, log on to https://www.editorialmanager.com/pone/ and select the 'Submissions Needing Revision' folder to locate your manuscript file.

Please include the following items when submitting your revised manuscript:

- A rebuttal letter that responds to each point raised by the academic editor and reviewer(s). You should upload this letter as a separate file labeled 'Response to Reviewers'.
- A marked-up copy of your manuscript that highlights changes made to the original version. You should upload this as a separate file labeled 'Revised Manuscript with Track Changes'.
- An unmarked version of your revised paper without tracked changes. You should upload this as a separate file labeled 'Manuscript'.

-->If you would like to make changes to your financial disclosure, please include your updated statement in your cover letter. Guidelines for resubmitting your figure files are available below the reviewer comments at the end of this letter.

If applicable, we recommend that you deposit your laboratory protocols in protocols.io to enhance the reproducibility of your results. Protocols.io assigns your protocol its own identifier (DOI) so that it can be cited independently in the future. For instructions see: https://journals.plos.org/plosone/s/submission-guidelines#loc-laboratory-protocols. Additionally, PLOS ONE offers an option for publishing peer-reviewed Lab Protocol articles, which describe protocols hosted on protocols.io. Read more information on sharing protocols at https://plos.org/protocols?utm_medium=editorial-email&utm_source=authorletters&utm_campaign=protocols.

We look forward to receiving your revised manuscript.

Kind regards,

Ricardo Q. Gurgel, PhD

Academic Editor

PLOS ONE

Journal Requirements:

1. If the reviewer comments include a recommendation to cite specific previously published works, please review and evaluate these publications to determine whether they are relevant and should be cited. There is no requirement to cite these works unless the editor has indicated otherwise.

2. Please review your reference list to ensure that it is complete and correct. If you have cited papers that have been retracted, please include the rationale for doing so in the manuscript text, or remove these references and replace them with relevant current references. Any changes to the reference list should be mentioned in the rebuttal letter that accompanies your revised manuscript. If you need to cite a retracted article, indicate the article’s retracted status in the References list and also include a citation and full reference for the retraction notice.

[Note: HTML markup is below. Please do not edit.]

[NOTE: If reviewer comments were submitted as an attachment file, they will be attached to this email and accessible via the submission site. Please log into your account, locate the manuscript record, and check for the action link "View Attachments". If this link does not appear, there are no attachment files.]

To ensure your figures meet our technical requirements, please review our figure guidelines: https://journals.plos.org/plosone/s/figures

You may also use PLOS’s free figure tool, NAAS, to help you prepare publication quality figures: https://journals.plos.org/plosone/s/figures#loc-tools-for-figure-preparation.

NAAS will assess whether your figures meet our technical requirements by comparing each figure against our figure specifications.

# Author comment: Author response to Decision Letter 3

**DOI:** 10.1371/journal.pone.0354372.r006

4 Jul 2026

Dear Academic Editor,

We are deeply grateful for the invaluable comments and recommendations provided by you and the reviewers once again. Your insights have significantly improved the quality and relevance of our manuscript, and we have carefully addressed each point raised. We have highlighted the changes made in the revised manuscript and attached a point-by-point response to the reviewers' comments as requested. Your expert guidance and dedication throughout the review process have been instrumental in enhancing the overall rigor of our work.

Thank you for your thoughtful review and support.

Warm regards,

Selemaye Zenebe, corresponding author

Arba Minch University, selemayez@gmail.com

Changes were made to the reference list based on the suggestions and comments from the editor and reviewers

Retracted references that are removed from the manuscript include:

1. Cancer WIAfRo. Cancer Tomorrow: estimated number of cervical cancer incidence and mortality in Ethiopia.

2. Organization. WH. Ethiopia launches Human Papillomavirus Vaccine for 14-year-old Girls, 2018 World Health Organization. 2021

Newly added 8 relevant references to the manuscript:

1. Reference 4. Teklehaimanot DA, Mekuria AD, Dadi AF, Derseh BT. Precancerous lesion determinants in women attending cervical cancer screening at public health facilities in North Shoa Zone, Amhara, Ethiopia: an unmatched case-control study. BMC Women's Health. 2024;24(1):271.

2. Reference 5. Tesfaye E, Kumbi B, Mandefro B, Hemba Y, Prajapati KK, Singh SC, et al. Prevalence of human papillomavirus infection and associated factors among women attending cervical cancer screening in the setting of Addis Ababa, Ethiopia. Scientific Reports. 2024;14(1):4053.

3. Reference 6. Temesgen MM, Alemu T, Shiferaw B, Legesse S, Zeru T, Haile M, et al. Prevalence of oncogenic human papillomavirus (HPV 16/18) infection, cervical lesions, and their associated factors among women aged 21–49 years in Amhara region, Northern Ethiopia. 2021;16(3):e0248949.

4. Reference 11. Shapiro GKJCO. HPV vaccination: an underused strategy for the prevention of cancer. 2022;29(5):3780-92.

5. Reference 16. Enyew EB, Kasaye MD, Kebede SD, Feyisa MS, Serbessa NG, Tebeje TM, et al. Human Papillomavirus Vaccination uptake and associated factors among school girls aged between 9–14 years in Ethiopia: Performance Monitoring for Action (PMA-ET) 2023, multilevel analysis. 2025;20(6):e0325557.

6. Reference 19. Rincon NL, McDowell KR, Weatherspoon D, Ritchwood TD, Rocke DJ, Adjei Boakye E, et al. Racial and ethnic disparities in human papillomavirus (HPV) vaccine uptake among United States adults, aged 27–45 years. 2024;20(1):2313249.

7. Reference 24. Aguboshim FCJWJoAR, Reviews. Adequacy of sample size in a qualitative case study and the dilemma of data saturation: A narrative review. 2021;10(3):180-7.

8. Reference 25. Naeem M, Ozuem W, Howell K, Ranfagni SJIJoQM. Demystification and actualisation of data saturation in qualitative research through thematic analysis. 2024;23:16094069241229777.

Response to reviewer #1 comments

Comment 1: Your data statement still reads “Yes - all data are fully available without restriction”. Then, on page 16, line 445, you state: “Data availability: All important data were available with a reasonable request from the corresponding author.” These are contradictory. Please update

Response 1: Thanks for your comment. The sentence is corrected to “All important data were available within the manuscript.” On page 17, Line 463

Comment 2: I’m not sure that you did this. The text now reads: “Cervical cancer (CC) is the fourth most frequently diagnosed and fourth most common cause of 64 cancer-related mortality, accounting for 604,127 new cases and 341,831 deaths globally in 2020.”

• The source you cite states: “Cervical cancer is the fourth most frequently diagnosed cancer and the fourth leading cause of cancer death in women, with an estimated 604,000 new cases and 342,000 deaths worldwide in 2020 (Table 1, Fig.

The words are very similar, but the meaning is very different, as this is in women. What you have written is incorrect.

Response 2: Thank you for your feedback. The sentence is updated accordingly on page 3, line 63.

Comment 3: ----Update: ---

There are issues with the quality of the English grammar. ‘Purposive sampling’ is not correct English. ‘Conveniently recruited’ is not correct English. You could state, e.g., ‘we used purposive sampling’ or ‘purposive sampling was used’. Equally, ‘We used convenience sampling’ or ‘convenience sampling was used’.

Response 3: Thank you for your feedback. It was updated on page 5, line 136 and line 138, and page 7, line 203.

Comment 4: ----Update: ---

• The text now states: “Parents' interviews were categorized into two major themes based on the objectives of the study.” So were some interviews in just one category? Were some in both? Do you mean coded? Categorization implies that participants were not in both categories.

Response 4: Thank you for your valuable comment. We have updated it on Page 8, Line 217

Response to reviewer #2 comments

ABSTRACT

Comment 1: Lines 49 to 50- Perceived facilitators and barriers did not emerge as themes because the authors already stated those as their objective. Alternative wording could be, “findings were split between two broad themes…”

Response 1: Thank you for your comment. The Sentence was changed to “Findings were split between two major themes: perceived facilitators and barriers to human papillomavirus vaccine acceptance.” on page 2, line 49 – 50

Comment 2: Line 66- remove “being” in “being located”

Response 2: Thank you for your comment. “Being” is removed from “being located” on page 3, line 66.

Comment 3: Line 71- mention the well-known risk factors

Response 3: Thank you for your constructive comment. The well-known risk factors are mentioned on Page 3, lines 72-74.

Comment 4: Line 80- Cite the first sentence.

Response 4: Thank you for your comment. The first sentence is cited appropriately on page 4, line 83.

Comment 5: Line 97- “Recent studies show that…”-citation should be more than 1 based on this statement.

Response 5: Thank you for your comment. More than one study is cited based on the suggestion on page 4, lines 101 and 102.

Comment 6: Lines 106 to 107- Stating there are no studies is a pretty strong statement and subjective. A better alternative could be to state something along the lines of “to the nest of our knowledge, no studies have…”

Response 6: Thank you for your comment. Updated according to the comment on Page 4, line 110.

METHODS

Comment 7: Include a conceptual foundation or paradigm on which your study is based - such as critical realist, constructivist, grounded theory, etc.

Response 7: Thank you for your insightful recommendation. We agree that a conceptual foundation can give qualitative research a crucial perspective. In this instance, we have purposefully chosen a basic descriptive approach, concentrating on identifying attitudes, enablers, and obstacles to HPV vaccine use among rural parents in the Alle district. We see this descriptive design and content analysis as a first step to lead to deeper exploration in the future using other qualitative methodologies.

Please let us know if further clarification would be helpful.

Comment 8: Lines 137 to 138-include citation

Response 8: Thank you for your comment. Citation added to it on page 5, lines 142 &143.

RESULTS

Comment 9: Line 287- Change to “Other participants also expressed”

Response 9: Thank you for your comment. “Another participant also expressed” changed to “Other participants also expressed” on Page 11, line 292, according to the comment.

Comment 10: Sub-themes on misconceptions that have quotations about fertility should be added to “fear of side-effects” or vice versa, depending on the entire context of participants’ accounts. It is a bit unclear how the quotations on fertility differ between these 2 sub-themes.

Response 10: Thank you for the comment. Concerning the possible overlap between the sub-themes "Misconceptions" and "Fear of Side Effects," especially about participant quotes about fertility, we value your remark. It is sometimes ambiguous to separate themes, but, in our study, under the misconceptions sub-theme, we have identified comments about fertility that contain medical misinformation such as "the vaccine makes girls infertile" and we have grouped quotes that convey general worry or fear of unidentified side effects under fear of side effects, and have not included those here that contain false information.

DISCUSSION

Comment 11: The authors provided a summary of the results in the discussion and did not do much of a discussion. Studies cited were not expanded on how they differed or aligned with the current study. Implications of the findings, thoughts around why the results were what they were, should be discussed, backed by past literature (whether for or against). Of value could be other studies in rural areas that have examined HPV vaccination-whether quantitative or qualitative, and studies abroad, e.g. US, can also be cited.

Response 11: Thanks for your comment. We value your comment, and we have updated parts of the discussions again.

Comment 12: Lines 400, 403 to 404- include a summary of the studies mentioned.

Response 12: Thank you for your comment. The summary of the studies is added on page 15, lines 409-4012, and lines 415-417.

CONCLUSION

Comment 13: The authors should also mention something about leveraging parents’ perceptions about the benefits. Pushing that messaging forward could also be helpful, as those were found to be directly correlated with vaccine acceptance.

Response 13: Thank you for your suggestion. Revised based on the comment on page 16, lines 448-452.

Attachment Submitted filename: Response_to_Reviewers_auresp_3.docx

# Editor report: Decision Letter 3

**DOI:** 10.1371/journal.pone.0354372.r007

**Authors:** Ricardo Gurgel

8 Jul 2026

Perceived facilitators and barriers against human papillomavirus vaccine among rural parents with eligible daughters in the Alle district, Southern Ethiopia.

PONE-D-24-58812R3

Dear Dr. Zenebe,

We’re pleased to inform you that your manuscript has been judged scientifically suitable for publication and will be formally accepted for publication once it meets all outstanding technical requirements.

Within one week, you’ll receive an e-mail detailing the required amendments. When these have been addressed, you’ll receive a formal acceptance letter and your manuscript will be scheduled for publication.

An invoice will be generated when your article is formally accepted. Please note, if your institution has a publishing partnership with PLOS and your article meets the relevant criteria, all or part of your publication costs will be covered. Please make sure your user information is up-to-date by logging into Editorial Manager at Editorial Manager® and clicking the ‘Update My Information' link at the top of the page. For questions related to billing, please contact billing support.

If your institution or institutions have a press office, please notify them about your upcoming paper to help maximize its impact. If they’ll be preparing press materials, please inform our press team as soon as possible -- no later than 48 hours after receiving the formal acceptance. Your manuscript will remain under strict press embargo until 2 pm Eastern Time on the date of publication. For more information, please contact onepress@plos.org.

Kind regards,

Ricardo Q. Gurgel, PhD

Academic Editor

PLOS One

Additional Editor Comments (optional):

Reviewers' comments:

# Editor report: Acceptance letter

**DOI:** 10.1371/journal.pone.0354372.r008

**Authors:** Ricardo Gurgel

PONE-D-24-58812R3

PLOS One

Dear Dr. Zenebe,

I'm pleased to inform you that your manuscript has been deemed suitable for publication in PLOS One. Congratulations! Your manuscript is now being handed over to our production team.

At this stage, our production department will prepare your paper for publication. This includes ensuring the following:

* All references, tables, and figures are properly cited

* All relevant supporting information is included in the manuscript submission,

* There are no issues that prevent the paper from being properly typeset

You will receive further instructions from the production team, including instructions on how to review your proof when it is ready. Please keep in mind that we are working through a large volume of accepted articles, so please give us a few days to review your paper and let you know the next and final steps.

Lastly, if your institution or institutions have a press office, please let them know about your upcoming paper now to help maximize its impact. If they'll be preparing press materials, please inform our press team within the next 48 hours. Your manuscript will remain under strict press embargo until 2 pm Eastern Time on the date of publication. For more information, please contact onepress@plos.org.

You will receive an invoice from PLOS for your publication fee after your manuscript has reached the completed accept phase. If you receive an email requesting payment before acceptance or for any other service, this may be a phishing scheme. Learn how to identify phishing emails and protect your accounts at https://explore.plos.org/phishing.

If we can help with anything else, please email us at customercare@plos.org.

Thank you for submitting your work to PLOS ONE and supporting open access.

Kind regards,

PLOS ONE Editorial Office Staff

on behalf of

Professor Ricardo Q. Gurgel

Academic Editor

PLOS One
